# E-Portfolios for self-regulated and co-regulated learning: A review

**DOI:** 10.3389/fpsyg.2022.1079385

**Published:** 2022-11-21

**Authors:** Ricky Lam

**Affiliations:** Department of Education Studies, Hong Kong Baptist University, Hong Kong, Hong Kong SAR, China

**Keywords:** e-Portfolios, writing, self-regulation of learning, co-regulation of learning, formative assessment

## Abstract

The reflective component of e-Portfolios is said to help students improve second or foreign language writing in terms of motivation and academic results. Despite this positive advocacy, scholars remain unclear about how e-Portfolios can develop students’ self-regulatory abilities in writing classrooms, especially when students engage in complex e-Portfolio construction processes with peers, parents, teachers, their community, digital tools, and online resources. Recently, researchers have argued that not only do e-Portfolios promote self-regulated learning, but they also support co-regulation of learning wherein the latter is socially mediated by curriculum design, instructional materials, and in-class interaction patterns. Indeed, students’ inner development of self-regulatory capacity is closely influenced by external forces, which deserve more scholarly investigation. The review fills this gap by emphasizing that besides self-regulated learning, e-Portfolios can support students’ co-regulation of learning by way of their connectivity, visibility, and circulation. This review has four sections. The first section defines key concepts, namely e-Portfolios, self-regulated, co-regulated, and socially shared-regulated learning, and introduces how e-Portfolios foster self-regulation of learning in second language writing. The second section unpacks two conceptual models that underpin self-regulated and co-regulated learning relating to e-Portfolios. The third section presents a brief review, showcasing how e-Portfolios featuring self-regulation of learning can also support co-regulation of learning. The final section recommends strategies that facilitate self-regulation and co-regulation of learning in e-Portfolios reciprocally, and discusses implications for pedagogy and research.

## Introduction

Since the late 1990s, alternative assessment has been extensively advocated. One of the prominent examples is portfolio assessment, be it in the paper-based or electronic medium (Yancey, 2019). At the same time, the majority of developed and developing countries have promoted e-learning as one of their key curricular reform initiatives. E-Portfolios, a natural successor of their print counterparts, have been burgeoning due to the fact they can easily be supported by Internet-enabled digital gadgets (mobile phones and tablet computers) and infrastructure (Wi-Fi connection) worldwide (Ibarra-Sáiz et al., 2020). In second language education, e-Portfolio implementations have begun to develop rapidly, particularly during the past fifteen years.

In this paper, e-Portfolios are defined as digital dossiers, in which learners create, curate, reflect upon, and disseminate their multimodal artifacts to document their language learning development (Yancey, 2009). E-Portfolio assessment serves the purpose of evaluating students’ writing formatively and summatively through digital portfolio tools, usually involving their active participation in self-regulation and co-regulation of iterative composing processes (i.e., self- and peer-assessment; Lam, 2021). Thus, self-regulation is about a psychological process where an individual exerts control on their behaviors intentionally, whereas self-regulation of learning, aka self-regulated learning, is situated in academic contexts, namely at all levels of formal education in second or foreign language learning (Teng and Zhang, 2022). Self-regulated learning alludes to how learners monitor their cognition, behaviors, motivation, and emotion to achieve personal goals with internal and external feedback (Clark, 2012). Co-regulated learning is about advancing one’s own self-regulated learning capacity by interacting with a more capable other, namely the teacher or high-performing peers. Socially shared-regulated learning is characterized as joint, co-constructed regulation by multiple learners to complete a group activity, a mini project, or an online collaborative writing task (Allal, 2020).

In e-Portfolio scholarship, there is no shortage of studies that investigated the effectiveness of e-Portfolios, used as a pedagogical and an alternative assessment method, on students’ second language learning, especially in writing (Barrot, 2021; Pourdana and Tavassoli, 2022). These studies primarily measured whether e-Portfolio interventions could improve students’ learning outcomes (scores or grades), self-efficacy beliefs, and reflective skills (capacity to self-reflect upon language learning through different e-Portfolio tools). Despite these findings, not much has been done to explore how and why e-Portfolios can help students develop self-regulatory processes in learning writing within a second language classroom context (Teng and Zhang, 2016, 2022). Furthermore, Allal (2020, 2021) argues that alternative assessments, like e-Portfolios, support not only self-regulated learning, but also co-regulated learning, in which the latter is socially mediated by curriculum design, instructional materials, and in-class interaction patterns in addition to each individual’s inner development of self-regulated ability. These two research agendas remain a road “less traveled” in e-Portfolio studies and, thus far, receive relatively less scholarly attention.

To fill these much-needed gaps, the current paper discusses how e-Portfolios can support self-regulated and co-regulated learning in second language writing under the aegis of theoretical models, empirical findings, and practical advice. After this introductory section, the paper unfolds two oft-cited self-regulated learning models that explain the interdependence between self-regulated and co-regulated learning in writing. Then, it reviews how e-Portfolios facilitate self-regulated and co-regulated learning in second language writing classrooms with state-of-the-art studies. Lastly, the paper recommends feasible classroom strategies to sustain co-regulated learning in e-Portfolio programs, and discusses implications for teaching and research when teachers plan to attempt the e-Portfolio tool.

## Conceptual models

In his seminal work, Panadero (2017) has reviewed six oft-cited models of self-regulated learning and proposed four future implications for research. Of these six models, I have chosen to discuss Zimmerman’s (2000) cyclical phase model of self-regulated learning and Hadwin et al.’s (2018) self-regulation model in collaborative contexts, namely self-regulation, co-regulation and socially shared-regulation owing to four reasons. First, they are considered two of the most cited self-regulated learning models in psychology scholarship. Second, the theoretical underpinnings of these two models align with the theme of this review paper. Third, these two self-regulated learning models can best represent the core attributes of e-Portfolios, including metacognition (curation of and reflection on digital artifacts to improve learning), collaboration (co-construction of new knowledge with peers and the teacher), and interactivity (use of online and communal resources; Lam, 2022). Fourth, the final reason why Zimmerman’s (2000) cyclical phase model was adopted is that its tripartite component, i.e., forethought, performance, and self-reflection, has dovetailed well with the pedagogical procedures of most strategy-based instruction in second language writing, namely goal-setting, active monitoring of progress, interpreting feedback for revision, and adjusting goal-related actions (cf. Andrade and Brookhart, 2016; Bonner and Chen, 2020; Teng, 2022).

As said above, Zimmerman’s cyclical phase model has forethought, performance, and self-reflection phases. Forethought entails task analysis, goal-setting, and selection of strategies to attain goals; performance involves metacognitive self-monitoring of writing development; and self-reflection encompasses self-judgment and self-adjustment, e.g., text revision (Chen and Bonner, 2020). These three phases of self-regulated learning are iterative, dynamic, and cyclical in nature. They are typically considered *internal* psychological processes, revealing how a learner controls their mind, actions, motive, and feelings when achieving a learning goal. Although Zimmerman’s model of self-regulated learning originates from socio-cognitive theory, his model mainly regards the learner’s language development as solitary endeavors, ignoring the fact that students do interact with others and are influenced by their immediate educational environments when learning second language writing (Bandura, 1986; Andrade and Brookhart, 2020). In e-Portfolio instruction and assessment, students are likely to perform self-regulated learning practices (i.e., self-assessment and self-reflection tasks) together with peers, caregivers, teachers, and other community members in order to solicit their assistance. Furthermore, students may employ a range of online resources and language learning strategies to compile and disseminate their digital artifacts collectively (Yancey, 2019).

Because of this, I turn to the second self-regulated learning model by Hadwin et al.’s (2011), Hadwin et al.’s (2018). In their model, self-regulated learning is contextualized in collaborative settings as well as social learning. As Panadero (2017, p. 15) pointed out, “group members need to share the regulation of their learning” rather than self-regulating their own learning. The essence of Hadwin et al.’s (2018) model lies in the fact that self-regulated learning should develop and combine personal and social processes, which align with a Vygotskian view of other-regulation (Allal, 2020). Self-regulated learning involves not only *self* but also *others* within a larger group (Thomas et al., 2021). Hadwin et al. (2011) explicated that three modes of regulation coexisted in their model interactively, namely self-regulation, co-regulation, and socially shared regulation. They further differentiated the three modes of regulation by stating that “co-regulation” was a transitional process of support by more capable others and/or by instructional and assessment tools that fostered development of individual self-regulation and of socially shared regulation when a learning task was carried out collaboratively by a group. Of these three modes, I predominantly discuss the first two, which constitute the central argument of this paper, but not the third, which remains relevant yet out of the scope of this brief report.

Hadwin et al. (2018, p. 5) viewed self-regulated learning in collaboration as “an individual learner’s self-regulatory actions used to interact with other group members”, whereas they defined co-regulated learning in collaboration as “affordances and constraints stimulating the (student’s) appropriation of strategic planning, enactment, reflection, and adaptation (occurring when in interaction with other students or group members)”. Hence, the former self-regulatory actions, even in the collaborative manner, remain internal and individual, but the latter co-regulatory actions seem to be either guided/directed by capable others (peers, parents, or the teacher) or contributed by one another equally. Co-regulatory actions also capitalize on utilizing other learners, group members, and external tools (e.g., classmates’ e-Portfolio artifacts, useful websites, cloud-based editing tools, online machine translation software, etc.) as digital resources to facilitate effective second language writing.

In brief, Hadwin et al.’s self-regulation model in collaborative settings highlights the important roles of context, peers, community members, external resources, and digital tools, which not only enhance individual self-regulation, but also promulgate the joint influence of regulation of these human and material resources to support co-regulation of learning in certain academic settings, such as second language writing classrooms (Lam, 2015; Allal, 2021; Bowen et al., 2022). Thus far, not much has been done to understand how e-Portfolios, a promising technology-assisted learning tool, can support students’ development of self-regulated and co-regulated learning in writing instruction, especially how and the extent to which co-regulated learning (online peer assessment or collaborative writing tasks) exerts social influence on a learner’s mastery of self-regulated learning skills within a context of e-Portfolio pedagogy, namely active participation in a group mini-project leading to self-assessment of one’s own writing ability (Teng and Zhang, 2022).

## Review of evidence

This section reviews six empirical studies, and looks into whether utilizing e-Portfolio interventions to promote co-regulated learning may support students’ self-regulated learning in second language writing classrooms. The selection of these six e-Portfolio studies was retrieved from Lam’s (2022) recent review. Before I further fine-tuned the search procedures based upon this review, I have set two inclusion and two exclusion criteria. The inclusion criteria comprise: studies featuring a component of strategy-based instruction, and studies adopting either a process or genre-based pedagogical approach. The exclusion criteria entail: review studies with no empirical data and studies having no mention of self-regulated learning. Even with the presence of these pre-selected criteria, it is not possible to incorporate all relevant e-Portfolio studies with a focus on self-regulated learning into this brief report, given that its scope and depth remain limited. Also, as not all could be included in the current study, I chose those emphasizing a strong interconnection between self- and co-regulation of learning in academic writing contexts. The six studies are arranged and presented in chronological order.

Nicolaidou (2013) introduced weblogs (i.e., WordPress) as an e-Portfolio tool in one Cyprus primary school classroom. Following a pre-test and post-test design, the study intended to identify whether weblog-based e-Portfolios could improve primary pupils’ writing performance, namely accuracy and peer feedback skills. The researcher adopted a process writing approach in the weblog-based e-Portfolio program by encouraging self-regulated and co-regulated learning with active use of self, peer, and teacher feedback as online resources to upgrade student writing performance. Quantitative data showed that the student informants had marked improvement in their web-blog entries. One intriguing finding was that most informants were able to detect peers’ errors and provide them with direct and indirect written corrective feedback accurately. This heightened language awareness (an outcome of co-regulated learning) contributed enormously to an increase in their post-writing test scores (a testimony to the development of self-regulated learning capacity). This finding is consistent with that of Allal’s (2021) and Yan’s (2020) studies, where formative co-regulation of writing had a role to play in facilitating self-regulation of writing, namely self-assessment and text revision. Another finding of the study indicated that weblog-based e-Portfolios could enhance co-regulation of learning in writing *via* active participation in online peer assessment.

Aydin (2014) investigated 101 English-as-a-foreign-language (EFL) writers’ attitudes toward adopting a Facebook-based e-Portfolio tool (i.e., F-Portfolio) and their conceptions about its affordances and constraints. The informants’ attitudes and perceptions regarding the F-Portfolio instruction were measured by four questionnaires, namely a baseline questionnaire, the F-Portfolio Attitude Scale, the Portfolio Contribution Questionnaire, and the Portfolio Problem Questionnaire. The first questionnaire was open-ended, whereas the other three employed 5-point Likert scale items. Findings revealed that F-Portfolios helped improve students’ reading, writing, vocabulary, and research skills. Nevertheless, the informants considered F-Portfolio compilation processes uninteresting, cumbersome, and time-consuming, although they were positive about this alternative writing instruction. Another surprising finding was that the students perceived F-Portfolios as being less user-friendly to promote self-regulated learning in writing, because the platform’s interface could not facilitate online composing of interim drafts. Additionally, the informants found it taxing to provide synchronous peer feedback on F-Portfolios, because other students might not be online simultaneously, especially when most informants spent fewer than 3.8 h per week to compile their portfolios. Hence, co-regulation of learning was somewhat difficult to take place, since peer learning was neither supported by F-Portfolio software nor by its instructional design (i.e., F-Portfolios being a close system not open to external members).

Lam (2015) examined how explicit strategy instruction could enhance EFL students’ metacognitive knowledge and development of self-regulated learning. In a print portfolio program, the author implemented a 15-week process writing approach, which intended to equip four informants with metacognitive knowledge and self-regulated learning strategies to cope with academic writing tasks. All four informants, except one, showed moderate success of mastering metacognitive knowledge, since they were motivated to perform redrafting and text revisions. These four informants also developed a heightened sense of audienceship, enhanced text knowledge, and proficient use of diverse writing strategies to perform challenging portfolio tasks in the course. The explicit strategy instruction enabled lower-intermediate students to improve self-efficacy beliefs and self-regulated learning skills. One observation from the data sets revealed that the students still needed regular peer support and teacher scaffolding at various stages of the revising processes (an example of the role of peer and social regulation). This finding is compatible with what Allal (2021), Bowen et al. (2022), and Teng (2022) have discussed. These researchers have verified the effectiveness of explicit strategy instruction in first language and EFL writing contexts, and corroborated how co-regulation of learning could support the development of an individual student’s self-regulated learning in writing.

Bader et al. (2019) examined students’ perceptions of formative feedback used in writing portfolios. Forty pre-service teacher informants of EFL joined the study. 128 reflection notes were collected and coded. Four emerging themes were identified, including (a) learning from portfolio assessment; (b) learning from and attitudes toward teacher feedback; (c) learning from and attitudes toward peer feedback; and (d) strategies for acting upon feedback. The informants were generally positive about writing portfolios. They praised the usefulness of teacher and peer feedback, but considered teacher feedback more useful than peer feedback. It was because the former was reliable and honest, whereas the latter was perfunctory and less constructive (mostly friendly marking). Furthermore, the findings revealed that co-regulation of learning could be made effective when the instructors provided formative feedback at various junctures of the portfolio assessment process to assist students in self-reflecting, revising, and re-submitting assignments for summative grading. This finding is consistent with that of Xiao and Yang’s (2019) study regarding how teacher scaffolding could facilitate uptake of self-regulated learning strategies. In writing portfolios, the informants viewed peers as a learning resource albeit less trustworthy, which could help them develop self-regulated learning capacity *via* co-constructing active membership in diverse group activities.

Employing a quasi-experimental design, Barrot (2021) explored whether students had gains in writing performance after exposure to a Facebook-based e-Portfolio intervention in a Philippine university. Barrot adopted a pre/post-writing test design used in his previous work (see Barrot, 2018) to measure students’ writing ability. Introduced in the treatment group, the Facebook-based e-Portfolio program included eight stages, namely preparation, modeling, planning, collaborative writing, independent writing, revising, editing, and publishing. Findings showed that the treatment group (Facebook-based e-Portfolios) outperformed the control group (conventional paper-based portfolios) in the post-writing test scores. Based upon these quantitative data, Facebook-based e-Portfolios could create positive impacts on university students’ writing performance. The author explained that exposure to social pressure motivated the students to produce better written work, since they no longer wrote for the teacher only. Interestingly, he reported that the students became more self-regulated in learning writing, because they had ample opportunities to collaborate with peers and to learn how to improve their own drafts from their classmates’ perspectives. The development of internal self-evaluative judgment (an example of self-regulated learning) capitalized on co-regulation of learning promoted in Facebook-based e-Portfolios, such as peer editing, collaborative writing, and mini-research tasks.

Pourdana and Tavassoli (2022) investigated the impacts of e-Portfolios on EFL students’ modes of engagement in descriptive and narrative genres of writing, and whether the informants had improvement after joining this six-week genre-based e-Portfolio program. Utilizing a pre-test and post-test design, the researchers divided the informants (*n* = 56) into two groups, one who composed narrative writing tasks and the other, descriptive writing tasks. Research instruments included pre-test and post-test summative assessments, a tried-and-tested rubric, and the informants’ reflection logs. Findings revealed that e-Portfolios had greater impacts on lower-level writing skills (mechanics) than higher-level writing skills (organization). Quantitative findings showed that e-Portfolios could enable the informants to complete essays in both written genres satisfactorily. Concerning behavior engagement in e-Portfolios, the informants revised their interim drafts proactively and independently, implying that they were earnestly engaged in self-regulated learning due to the e-Portfolio curriculum. As to cognitive engagement, surprisingly, the informants did not agree on parts of teacher feedback, given that it deviated from their own self-assessments. This intriguing result may be interpreted as a situation where co-regulation of learning in e-Portfolios can positively support the development of self-regulated learning, e.g., fostering of one’s critical self-assessment skills as reported in Panadero et al.’s (2018) and Yan’s (2020) studies.

Overall, the findings of the reviewed studies indicated that print and electronic portfolios could help develop students’ self-regulation of learning in terms of improved post-writing test scores, enhanced audience awareness, academic writing performance (e.g., improved fluency), and peer feedback skills. More significantly, these portfolio programs enhanced co-regulation of learning through online peer assessment, planned teacher scaffolding, and students’ proactive engagement in e-Portfolio compilation processes, such as curation of works-in-progress, reflection upon artifacts, co-construction of scoring rubrics with peers/teachers among other aspects. Despite these unwritten benefits of e-Portfolios, the authors of these studies primarily regarded self-regulated learning in e-Portfolios as an *individual* academic endeavor, neglecting how other human or non-human resources, like teachers, instructional approach, types of digital tools, and curriculum design would play a part in enabling student development of self-regulated and co-regulated learning capacity concurrently. In fact, Andrade and Brookhart (2020) have advocated the idea of interdependence between student self-regulation and co-regulation in academic writing contexts, since regulation of one’s learning is socially mediated and becomes an indispensable feature of formative classroom-based assessment that supports multimedia collaborative learning in second language writing settings (Li, 2021; Cheung, 2022).

Based upon the above evidence, three emerging themes were identified to reveal the success of student development of co-regulated and self-regulated learning in respective e-Portfolio programs. They included: (1) process-oriented writing instruction; (2) opportunities for peer assessment; and (3) choice of e-Portfolio tools. For (1), all six studies adopted the process writing approach, in which the authors found it productive to initiate co-regulation of learning practices through planning, multi-drafting, self-assessing, and revising collaboratively, except for one study (i.e., Aydin, 2014). These cognitive composing strategies served as additional online resources to enable students to self-monitor and self-adjust their writings in order to close learning gaps. Also, the process approach facilitated reflective thinking and evaluative judgments that support co-regulation and self-regulation of learning reciprocally (Nicol et al., 2019; Ryan et al., 2021). With that said, teacher scaffolding was still needed amid this learner-centered approach, especially for young learners with limited exposure to self-regulated learning instruction.

For (2), it appears that the six portfolio-based programs included peer assessment as one form of instructional activity, although they all aimed at investigating students’ internal self-regulatory abilities. Albeit less reliable and moderately revisable as compared to teacher feedback, peer feedback was usually considered a catalyst to promote peer learning. It was somewhat convenient for students to provide and act upon online peer feedback synchronously and asynchronously. Providing opportunities for peer assessment is an important component of co-regulation of learning (Lam, 2015). Changing student mindsets about the usefulness of online peer feedback was also crucial, as some student informants still had bias against peer feedback. For (3), the selection of an appropriate e-Portfolio tool may affect the uptake of self-regulated and co-regulated learning skills. While it was common to use social media as an e-Portfolio platform, some informants found it demanding to manipulate the interface of Facebook, which did not technologically support self-reflection and provision of peer feedback (Aydin, 2014). In contrast, weblog-based e-Portfolios (i.e., WordPress or Wix) appear to be more suitable to facilitate development of literacy and co-regulated learning skills such as social learning, because these digital tools were in sync with the development of composing, editing, and revising skills collaboratively in a virtual learning environment (Nicolaidou, 2013).

## Recommendations and implications

This concluding section suggests how teachers can augment the implementation of self-regulated and co-regulated learning mutually by using self-assessment, and discusses the implications of how co-regulated learning promoted *via* e-Portfolios can influence students’ development of self-regulated learning capacity at various educational levels.

For a long time, professional literature has emphasized the effectiveness of self-assessment, particularly when applied in print and electronic portfolio environments (Lam, 2022). Self-assessment is considered part of self-regulated learning processes and a key component in e-Portfolio compilation journeys. Given its instructional role in second language writing contexts, scholars have claimed that self-assessment is a twenty-first century study skill learners should possess (Clarke and Boud, 2018). It is neither an option nor a luxury, but essential in writing instruction. Scholars further advocate that self-assessment should be introduced as early as possible in schooling, so that students are more likely to become life-long and self-regulated learners (Graham and Alves, 2021). In language assessment scholarship, self-assessment is a core element of formative assessment practices, which serve as a metacognitive tool, a learning-oriented task, and a process writing pedagogy (Clark, 2012; Panadero et al., 2019). Empirical evidence has corroborated that self-assessment can enhance student self-regulated learning capacity when students engage in academic writing tasks reflectively (Mazloomi and Khabiri, 2018).

More recently, Yan (2020) found that self-assessment could take place in all three phases of Zimmerman’s (2000) cyclical model of self-regulated learning (i.e., forethought, performance, and self-reflection), because “self-assessment is an ongoing practice across the whole self-regulated learning process, rather than a one-off action occurring at a particular time point” (p. 233). Although students set goals/analyze tasks in the forethought phase and monitor learning/select effective strategies in the performance phase, they remain proactive and reflective in performing self-assessment and then developing self-regulated and co-regulated learning skills correspondingly. According to Yan (2020), self-assessment should be treated as a scaffold and a learning-oriented tool. That said, self-assessment needs to be complemented by other inputs, including peer regulation (*via* peer assessment) and teacher direct instruction.

For instance, in Teng’s (2022, p. 7) study, the intervention program included teacher scaffolding at various junctures of the explicit writing strategy pedagogy, namely Stage 2: teacher-led discussion, followed by Stage 3: shared instruction (modeling), and Stage 5: scaffolding (guided practice with peer collaboration) before her informants composed and applied those learning strategies independently in Stage 6. An inclusion of scaffolding in self-assessment was also evident in Allal’s (2021) study that formative co-regulation (whole-class discussions on how to produce and revise the target genre) helped enhance fifth- and sixth-graders’ self-regulated learning in first language writing (improved writing quality in drafts and revisions). Despite this positive testimony, Fukuda et al. (2022) claimed that students, more often than not, did not have sufficient interactions with peers while developing their self-regulated learning skills. They recommended that co-regulation of learning in technology-assisted learning environments (e.g., e-Portfolios) should play an indispensable role in promoting the uptake of self-regulated learning strategies and self-assessment skills.

Lastly, I would like to discuss the implications of using e-Portfolios to support co-regulated and self-regulated learning in second language writing settings. First and foremost, self-regulated learning is best viewed not just as a lone, individual, and internal process, but is always linked to external processes where dynamic classroom dialogues and virtual interactions with peers, teachers, parents, and community members should be highlighted (Thomas et al., 2021). In fact, self-regulated learning is regarded as *self-as-others* or *self-with-others* learning processes that support both other-regulation and self-regulation of learning, especially within an e-Portfolio context featuring high connectivity. After all, e-Portfolios are interactive, integrative, and reflective in nature, which support both individual and collaborative writing, interdisciplinary knowledge transfer, and reflective thinking of collective goals in most second language writing curricula (Yancey, 2019). For instance, in primary and secondary classrooms, portfolio tasks could include guided practice elements, such as box-ticking checklists, reflective journals with prompts and teacher commentary, and two-stage assignments with opportunities for revision using teacher feedback. Tasks with regular “check-point” systems are equally beneficial, given that learners, especially for those younger and less capable ones, need more ongoing support if they want to develop self- and co-regulated learning skills. In university classrooms, portfolio tasks could be designed as collaborative and inquiry-based as possible with minimal teacher scaffolding, so that advanced learners can develop communication, problem-solving, and critical thinking skills collectively and then individually.

Besides adopting common learning management systems (weblogs or Moodle), the use of relatively up-to-date digital tools or social media platforms to support co-regulation and self-regulation of learning has become a forthcoming trend, namely, Facebook-based, Instagram-based, or TikTok-based e-Portfolios. It is because these platforms are user-friendly, accessible, free-of-charge, synchronous, and reciprocal (especially those with online chat functions), they are welcomed by most secondary and university students who already use these social networking sites in daily lives. Nevertheless, focused training in how to manipulate the functionalities of these digital tools to archive and showcase academic writing as part of e-Portfolio contents should be provided for the students, since not everyone is proficient in using these tools for the academic purpose (Aydin, 2014). For primary school learners, the use of customized e-Portfolio tools (e.g., FreshGrade) appears to be more appropriate as its template is uniquely designed for younger learners who only have limited digital competence and keyboarding skills. Although social media tools are not pedagogically designed to promulgate co-regulation of learning, these tools can unquestionably increase students’ online presence by actively involving them in compiling, curating, and reflecting upon multimedia artifacts to achieve their writing goals (Lam and Moorhouse, 2022).

Other than peer learning, researchers may consider exploring the extent to which and how other contextual factors/interventions may play a part in facilitating or impeding co-regulation and self-regulation of learning mutually *via* e-Portfolios, namely e-Portfolio task design, explicit strategy-based instruction, and student enactment with multisource e-feedback. If task designs are iterative involving use of peer feedback to influence creation of self-feedback or vice versa such as peer critique with rationalizations, university students are likely to develop a heightened metacognitive awareness, since such a formative assessment task involves deep reflection of peers’ and one’s own writing simultaneously (Farahian et al., 2021). Explicit strategy-based instruction enables students to develop self-reflective capacity by interacting with peers, teachers, and other community resources to achieve the learning goals (Teng, 2022). This instructional approach is useful for primary and secondary learners who need additional input from more capable others in second or foreign language classrooms. Providing timely and revisable e-feedback is only the first step. Teachers need to ensure how to harness effective feedback enactment, particularly of cognitive and behavioral ones to equip students with self-regulated learning skills (Fukuda et al., 2022). One practical strategy to maximize feedback enactment is that teachers may consider giving primary and secondary students direct written feedback with examples, so that they can acquire the correct linguistic form in their future writing. In university classrooms, teachers may consider providing advanced learners with indirect written feedback with clues in order to encourage them to fix their errors independently.

E-Portfolios are only a means to an end in achieving effective second language writing instruction. This technology-assisted tool is not a panacea, which could automatically facilitate co-regulation and self-regulation of learning in writing when student and teacher digital literacy remains low and the digital divide remains a cause for concern in certain educational contexts. To this end, teachers and researchers may explore how regulation *via* other contextual factors, such as consolidation of e-Portfolio curricula (multidisciplinary ones), teacher instructional approaches (use of flipped classrooms), and student willingness to enact e-feedback (learner agency in feedback process) can support co-regulated and self-regulated learning interdependently.

## Author contributions

RL was solely responsible for conceptualizing, drafting, editing, and revising the manuscript. This was his original work and developed from his recently funded project.
